# Association of 5α-Reductase Inhibitors With Dementia, Depression, and Suicide

**DOI:** 10.1001/jamanetworkopen.2022.48135

**Published:** 2022-12-22

**Authors:** Miguel Garcia-Argibay, Ayako Hiyoshi, Katja Fall, Scott Montgomery

**Affiliations:** 1 Clinical Epidemiology and Biostatistics, School of Medical Sciences, Faculty of Medicine and Health, Örebro University, Örebro, Sweden; 2 Department of Public Health Sciences, Stockholm University, Stockholm, Sweden; 3 Department of Epidemiology and Public Health, University College London, London, United Kingdom; 4 Public Health, Department of Social Medicine, Osaka University Graduate School of Medicine, Osaka, Japan; 5 Clinical Epidemiology Division, Department of Medicine, Karolinska Institutet, Solna, Sweden

## Abstract

**Importance:**

In recent decades, there has been increased interest in the possible adverse neurological effects of 5α-reductase inhibitors (5-ARIs), which have been used mainly for benign prostatic hyperplasia and androgenic alopecia. Numerous studies and reports have indicated associations of 5-ARIs with depression and suicide. However, most of these studies had methodological shortcomings, and very little is known about the potential association of 5-ARIs with dementia.

**Objective:**

To investigate the association of 5-ARI use with all-cause dementia, Alzheimer disease, vascular dementia, depression, and suicide.

**Design, Setting, and Participants:**

This Swedish register-based cohort study included 2 236 876 men aged 50 to 90 years between July 1, 2005, and December 31, 2018. Statistical analyses were performed from September 15, 2021, to May 25, 2022.

**Main Outcomes and Measures:**

A diagnosis of all-cause dementia, Alzheimer disease, vascular dementia, depression, or completed suicide.

**Exposures:**

A recorded prescription in the Swedish national prescription register of finasteride or dutasteride and duration of use.

**Results:**

Of 2 236 876 men (median age at the start of follow-up, 55 years [IQR, 50-65 years] and at treatment initiation, 73 years [IQR, 66-80 years]), 70 645 (3.2%) started finasteride treatment, and 8774 (0.4%) started dutasteride treatment. Men taking finasteride or dutasteride were at increased risk of all-cause dementia (finasteride: hazard ratio [HR], 1.22 [95% CI, 1.17-1.28]; dutasteride: HR, 1.10 [95% CI, 1.01-1.20]), Alzheimer disease (finasteride: HR, 1.20 [95% CI, 1.10-1.31]; dutasteride: HR, 1.28 [95% CI, 1.09-1.50]), vascular dementia (finasteride: HR, 1.44 [95% CI, 1.30-1.58]; dutasteride: HR, 1.31 [95% CI, 1.08-1.59]), and depression (finasteride: HR, 1.61 [95% CI, 1.48-1.75]; dutasteride: HR, 1.68 [95% CI, 1.43-1.96]). However, the magnitude of the association decreased over time, and the findings became statistically nonsignificant with continuous exposures over 4 years, except for depression, which showed a constant risk over time, with no differences between finasteride and dutasteride. In contrast, 5-ARIs were not associated with suicide (finasteride: HR, 1.22 [95% CI, 0.99-1.49]; dutasteride: HR, 0.98 [95% CI, 0.62-1.54]).

**Conclusions and Relevance:**

This cohort study found that, while men receiving 5-ARI treatment showed a higher risk for dementia in the initial periods after starting treatment, the decreasing magnitude of the association over time suggested that the risk may be, entirely or in part, due to increased dementia detection among patients with benign prostate enlargement. Both finasteride and dutasteride were similarly associated with depression with a constant risk over time, while neither drug was associated with suicide. Prescribing clinicians and potential users should be aware of the possible risks for depression associated with 5-ARI use.

## Key Points

**Question:**

Are 5α-reductase inhibitors (5-ARIs), used for benign prostatic hyperplasia and androgenic alopecia, associated with an increased risk for all-cause dementia, Alzheimer disease, vascular dementia, depression, and suicide?

**Findings:**

This register-based prospective cohort study in Sweden comprising 2 236 876 men found that, while 5-ARIs were associated with increased risk for all-cause dementia, Alzheimer disease, and vascular dementia after the start of treatment, the association became statistically insignificant over 4 years of exposure. There was no association between 5-ARIs and suicide, but both finasteride and dutasteride were associated with an increased risk for depression.

**Meaning:**

This study suggests that 5-ARIs might be associated with depression; in contrast, the decreasing trend in the magnitude of the associations between 5-ARIs and dementia seem to be due to increased detection of undiagnosed dementia, particularly at the early stage of 5-ARI treatment.

## Teaser

This population-based cohort study investigates the association of 5α-reductase inhibitor use with all-cause dementia, Alzheimer disease, vascular dementia, depression, and suicide among men in Sweden.

## Introduction

5α-Reductase inhibitors (5-ARIs), such as finasteride and dutasteride, are widely prescribed for the treatment of benign prostatic hyperplasia (BPH) and androgenic alopecia.^1^ Pharmacologically, 5-ARIs selectively inhibit the enzyme 5α-reductase (5-AR), which is involved in the conversion of testosterone into the androgen metabolite 5α-dihydrotestosterone (DHT) and expressed in many tissues, such as the prostate, testes, and hair follicles.^2^ Dutasteride and finasteride inhibit different 5-AR isozymes. Finasteride, 1 mg per day, selectively inhibits the type 2 5-AR isozyme, suppressing DHT by 70% in serum, whereas dutasteride, 0.5 mg per day, selectively inhibits the type 1 and type 2 5-AR isozymes, reducing DHT by more than 90%.^3^ Consequently, 5-ARIs achieve a good therapeutic response by decreasing DHT bioavailability and thus reducing its androgen-dependent association with the prostate and hair follicles.

In recent decades, clinical and public health concerns have arisen on the possible adverse neurological effects of 5-ARIs because low androgen levels have been potentially associated with elevated risk of cognitive decline^4,5^ and dementia.^6^ Studies have found that individuals taking 5-ARIs report adverse cognitive outcomes, such as difficulty in maintaining attention, mental cloudiness, and memory impairments.^7,8^ A register-based study reported an association between 5-ARIs and dementia during the initial 2 years of treatment,^9^ with a similar risk between finasteride and dutasteride. Several studies have found that 5-ARIs are associated with an increased risk for depression^8,10,11,12,13^ and suicide,^12,14,15,16^ although null associations have also been reported.^11,15,17^ A hypothesized mechanism to explain 5-ARI–induced depression and adverse cognitive outcomes is a decrease in the synthesis of neurosteroids and neuroactive steroids caused by the inhibition of 5-AR when taking 5-ARIs,^18^ owing to the important role that neurosteroids and neuroactive steroids play in neuroprotection, memory, and mood.^19^ However, many of the studies that investigated the association of 5-ARIs with dementia and depression had methodological shortcomings, including limited confounder control and small sample sizes. Furthermore, follow-up periods in the existing studies were short; thus, associations beyond 2 years of exposure to 5-ARIs remain unclear.

The aim of this study was to (1) investigate the association of 5-ARI use with all-cause dementia, Alzheimer disease, vascular dementia, depression, and completed suicide; (2) evaluate whether there are differences between finasteride and dutasteride in any of the outcomes; and (3) assess whether longer periods using 5-ARIs are associated with these outcomes by using large population-based national register data. We hypothesize an increased risk of dementia and depression that increases over time of exposure for individuals receiving 5-ARI treatment. Furthermore, given the larger inhibition of DHT with dutasteride, we hypothesize that dutasteride will show an increased risk of these outcomes compared with finasteride. In contrast, we do not expect to see an increased risk of suicide among individuals taking 5-ARIs.

## Methods

The study had ethical approval from the Regional Ethical Review Board in Stockholm, Sweden. Requirement for informed consent was waived for the present study because it was a secondary analysis of existing data. The investigation conforms to the 1964 Declaration of Helsinki and its later amendments or comparable ethical standards.^20^ This study followed the Strengthening the Reporting of Observational Studies in Epidemiology (STROBE) reporting guideline.

### Study Design and Population

We conducted a cohort study linking multiple Swedish national registers using the unique personal identity number issued to all residents.^21^ We identified 2 236 876 men who were alive, living in Sweden, and reached 50 to 90 years of age between July 1, 2005, and December 31, 2018. These individuals were followed up from either 50 years of age or July 1, 2005, whichever happened last, until diagnosis of each outcome separately, death, emigration, or December 31, 2018, whichever occurred first.

### Exposure

Using the National Prescription Register (PDR), we retrieved information on dispensed finasteride, dutasteride, and α-blockers; date dispensed; daily doses per package; and number of packages, and we created 2 types of time-varying exposure variables. One variable depicted the type of drug used at each time point, and the other variable represented the time under exposure (months 1-6, 7-12, 13-48, and ≥49; eMethods in Supplement 1). Individuals receiving 5-ARI treatment without a diagnosis of BPH were excluded.

### Outcomes

All-cause dementia was defined as (1) a recorded diagnosis of dementia (inpatient or outpatient specialist care services) or (2) a dispensed prescription for any of the following anticholinesterases: tacrine, rivastigmine, galantamine, memantine, or donepezil. The earliest date of either diagnosis or drug dispensation was used to define the incident date of dementia. Alzheimer disease, vascular dementia, and depression were defined as the first recorded primary diagnosis through inpatient or outpatient specialist care. Completed suicide was defined as a record in the National Death Register as the underlying cause of death (eTable 1 in Supplement 1 shows all *International Classification of Diseases* and Anatomical Therapeutic Chemical codes used to define each covariate). Individuals with an event before the start of follow-up were excluded (eMethods in Supplement 1).

### Covariates

The following conditions were included as time-varying variables in the model to control for the potential confounding effect of these risk factors^22,23^: obesity, eating disorders, hypertension, type 2 diabetes, lipid disorders, and use of β-blockers (eTable 1 in Supplement 1). If an individual had a recorded diagnosis of any of the covariates before the start of follow-up, the status of the given covariate started from 1, and 0 otherwise. We additionally adjusted for year of the start of follow-up to account for the increasing prescription trend for finasteride and dutasteride.

### Statistical Analysis

#### Main Analysis

Statistical analyses were performed from September 15, 2021, to May 25, 2022. For the descriptive analysis, individuals were classified as exposed to each drug if they ever took an α-blocker, finasteride, dutasteride, or a combination of a 5-ARI and α-blockers during follow-up. Characteristics of individuals were summarized by frequencies, proportions, and rates. For each drug, incidence rates (IRs) with 95% CIs were calculated fitting a quasi-Poisson generalized linear model with a log-link function to accommodate overdispersion in the outcomes. A Cox proportional hazards regression model with attained age as the underlying timescale was fitted to evaluate the association between 5-ARI exposure and each of our outcomes.

Using Cox proportional hazards regression models, we conducted 2 sets of analyses. First, to assess the overall association, an unadjusted model was fitted including a categorical variable with 5 levels (unexposed, finasteride, dutasteride, α-blockers, and combination of 5-ARIs and α-blockers). In the adjusted model, the following variables were included: year of start of follow-up, hypertension, obesity, type 2 diabetes, lipid disorders, and time exposed to each drug. The exposure variable and the covariates were allowed to vary over time. Therefore, the hazard at time *t* depends on the value of each covariate at that time point. Initially, each drug category (finasteride, dutasteride, α-blockers, and 5-ARI and α-blockers) was compared against unexposed individuals. We also performed comparisons of the 5-ARI drugs with α-blockers, as well as between finasteride and dutasteride. The Benjamini-Hochberg correction for multiple testing was performed. Second, to assess the association of time under exposure to 5-ARIs with outcomes, the variable representing the number of months of exposure, which divides person-time of exposed periods into 1 to 6 months, 7 to 12 months, 13 to 48 months, and 49 months or longer after 5-ARI treatment initiation, was used. Estimates were presented as hazard ratios (HRs) with 95% CIs. Data preparation and analyses were performed using R, version 4.0.4 (R Core Team).^24^ All *P* values were from 2-sided tests and results were deemed statistically significant at *P* < .05.

#### Sensitivity Analyses

First, to account for the delay between dementia onset and the actual diagnosis, and in line with previous research,^25^ analyses were repeated using the date of dementia onset defined as 3 years before the first diagnosis from the National Patient Register or prescription from the PDR. All individuals with an outcome before the start of follow-up were excluded. Second, we restricted 5-ARI users to those who started treatment at least 4 months after July 2005, when the PDR started, to avoid the inclusion of individuals with uncertain duration of 5-ARI exposure (21 792 5-ARI users after exclusions). Last, to reduce potential bias associated with differences in demographic and health characteristics, individuals receiving 5-ARI and α-blocker treatment were matched without replacement on the logit of the propensity score based on the aforementioned covariates (except β-blockers) in a 1:5 ratio. The start of follow-up for the matched controls was the same as for the index individuals. Individuals who ever took a combination of 5-ARIs and α-blockers were excluded. Analyses were rerun stratified by matching sets and adjusted for β-blockers.

## Results

Our cohort comprised 2 236 876 men, of whom a total of 79 227 (3.5%) men started 5-ARI treatment between January 1, 2005, and December 31, 2018 (Table 1). The median age was 55 years (IQR, 50-65 years) at the start of follow-up and 73 years (IQR, 66-80 years) at treatment initiation. During the follow-up, finasteride was prescribed to 70 645 men (3.2%), dutasteride to 8774 men (0.4%), and a combination of a 5-ARI with α-blockers to 121 409 men (5.4%). Men who ever received 5-ARIs were more likely to receive a diagnosis of hypertension or type 2 diabetes and receive β-blockers compared with those who never used 5-ARIs or α-blockers. Compared with those taking α-blockers, men receiving 5-ARI treatment were less likely to receive a diagnosis of type 2 diabetes, hypertension, obesity, or lipid disorders. Table 1 displays descriptive statistics of the cohort for each drug of interest.

**Table 1.** Descriptive Statistics of the Cohort Stratified by Drug Exposure During Follow-up

Characteristic	Individuals, No. (%)				
	Unexposed (n = 1 837 474)	Finasteride (n = 70 645)	Dutasteride (n = 8582)	α-Blockers (n = 198 766)	5-ARI and α-blocker (n = 121 409)
Age at death, median (IQR), y	80 (70-86)	86 (81-90)	85 (80-89)	82 (74-88)	84 (78-89)
Age first at 5-ARI medication, median (IQR), y	NA	75 (68-82)	74 (67-81)	NA	72 (66-78)
Continuous drug exposure, mo					
1-6	NA	70 645 (100)	8582 (100)	198 766 (100)	121 409 (100)
7-12	NA	50 540 (71.5)	6464 (75.3)	99 639 (50.1)	97 008 (79.9)
13-48	NA	45 856 (64.9)	5469 (63.7)	76 279 (38.4)	85 947 (70.8)
>49	NA	22 415 (31.7)	3333 (38.8)	35 091 (17.7)	39 347 (32.4)
Covariates					
β-Blocker use	585 598 (31.9)	36 156 (51.2)	4632 (54.0)	0	0
Type 2 diabetes	108 812 (5.9)	5885 (8.3)	741 (8.6)	19 645 (9.9)	10 141 (8.4)
Hypertension	123 811 (6.7)	7871 (11.1)	981 (11.4)	28 323 (14.2)	15 375 (12.7)
Obesity	17 075 (0.9)	427 (0.6)	41 (0.5)	2457 (1.2)	1037 (0.9)
Lipid disorder	13 958 (0.8)	661 (0.9)	76 (0.9)	2508 (1.3)	1332 (1.1)
Eating disorder	284 (0.02)	15 (0.02)	1 (0.01)	33 (0.02)	14 (0.01)
Outcomes					
Dementia	53 275 (2.9)	4774 (6.8)	644 (7.5)	5556 (2.8)	4199 (3.5)
Alzheimer disease	15 085 (0.8)	1209 (1.7)	196 (2.3)	1910 (1.0)	1395 (1.1)
Vascular dementia	10 504 (0.6)	972 (1.4)	135 (1.6)	1546 (0.8)	1172 (1.0)
Depression	64 269 (3.5)	2324 (3.3)	289 (3.4)	8585 (4.3)	4491 (3.7)
Completed suicide	5120 (0.3)	141 (0.2)	20 (0.2)	331 (0.2)	216 (0.2)

Abbreviations: 5-ARI, 5α-reductase inhibitor; NA, not applicable.

The overall IR of dementia was higher for finasteride users (IR, 79.3 [95% CI, 76.7-82.0] per 10 000 person-years) and dutasteride users (IR, 68.5 [95% CI, 63.0-74.3]) than unexposed individuals (IR, 22.0 [95% CI, 21.8-22.2]) or those taking α-blockers (IR, 35.9 [95% CI, 34.8-37.1]) (eTable 2 in Supplement 1). Such patterns were consistently observed in all outcomes, apart from depression, which showed a higher IR for those receiving α-blocker treatment alone (IR, 23.4 [95% CI, 22.5-24.3]) or in combination with 5-ARIs (IR, 21.3 [95% CI, 20.1-22.5]) compared with finasteride (IR, 18.8 [95% CI, 17.6-20.1]) and dutasteride (IR, 20.0 [95% CI, 17.1-23.3]). eTable 2 in Supplement 1 displays all IRs for each drug and outcome. The Figure shows the cumulative hazard function for each drug and outcome. Men taking finasteride or dutasteride were at increased risk of all-cause dementia (finasteride: HR, 1.22 [95% CI, 1.17-1.28]; dutasteride: HR, 1.10 [95% CI, 1.01-1.20]), Alzheimer disease (finasteride: HR, 1.20 [95% CI, 1.10-1.31]; dutasteride: HR, 1.28 [95% CI, 1.09-1.50]), vascular dementia (finasteride: HR, 1.44 [95% CI, 1.30-1.58]; dutasteride: HR, 1.31 [95% CI, 1.08-1.59]), and depression (finasteride: HR, 1.61 [95% CI, 1.48-1.75]; dutasteride: HR, 1.68 [95% CI, 1.43-1.96]) (Table 2).

**Figure.**
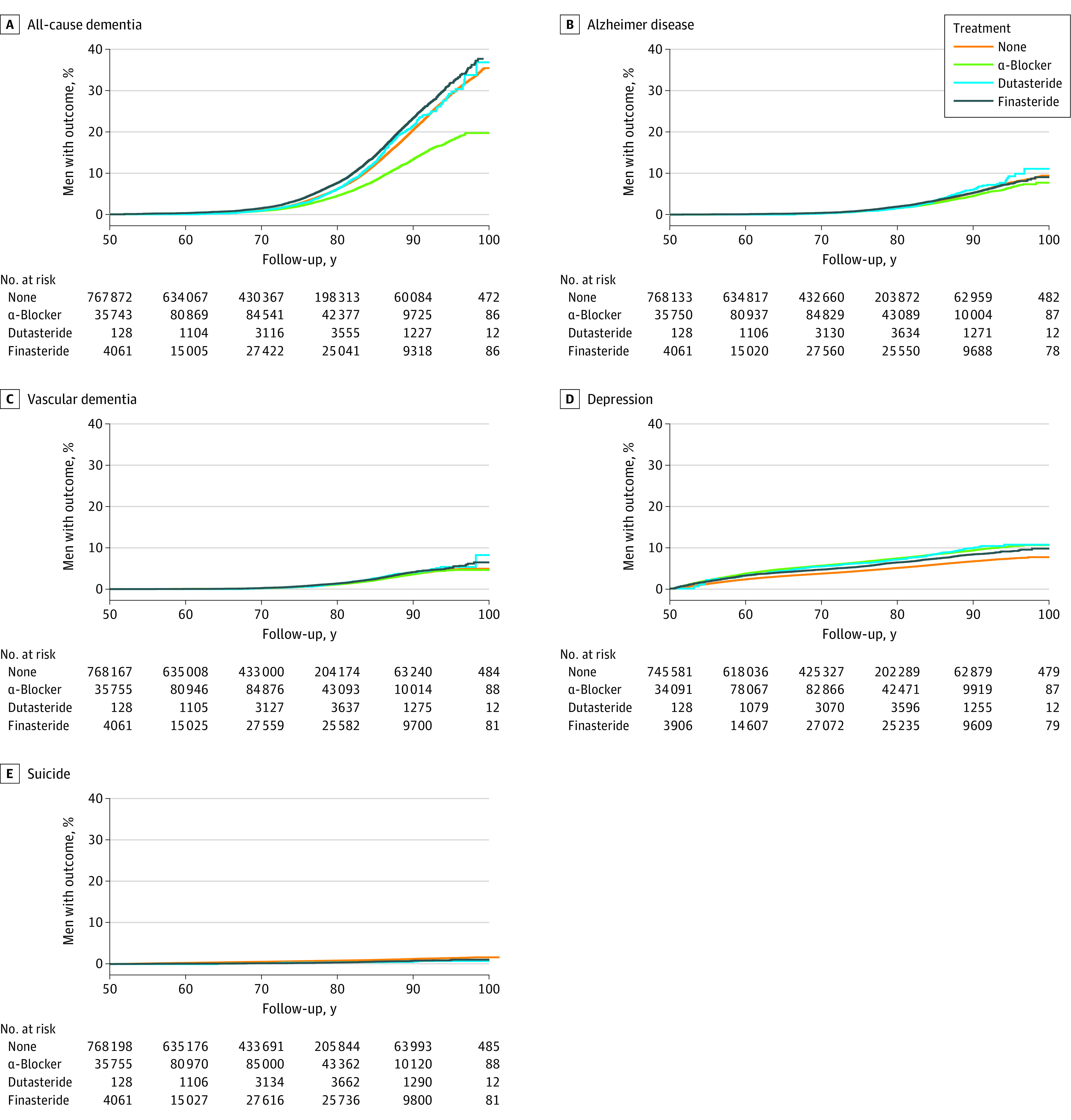
Cumulative Hazards of Outcomes for Unexposed Individuals and Those Receiving 5α-Reductase Inhibitor and α-Blocker Treatment

**Table 2.** Results From the Time-Varying Cox Proportional Hazards Regression Model Displaying the Association Between Each Type of Drug With Outcomes

Outcome and model	Finasteride		Dutasteride		α-Blockers		5-ARI and α-blockers	
	HR (95% CI)	*P* value	HR (95% CI)	*P* value	HR (95% CI)	*P* value	HR (95% CI)	*P* value
All-cause dementia								
Unadjusted	1.10 (1.06-1.14)	<.001	1.00 (0.92-1.09)	.96	0.82 (0.79-0.85)	<.001	0.81 (0.78-0.84)	<.001
Adjusted^a^	1.22 (1.17-1.28)	<.001	1.10 (1.01-1.20)	.04	0.71 (0.68-0.73)	<.001	0.73 (0.70-0.77)	<.001
Alzheimer disease								
Unadjusted	1.02 (0.95-1.09)	.65	1.11 (0.95-1.29)	.18	1.08 (1.02-1.14)	.01	1.02 (0.96-1.09)	.55
Adjusted^a^	1.20 (1.10-1.31)	<.001	1.28 (1.09-1.50)	<.001	1.10 (1.03-1.18)	.01	1.14 (1.04-1.25)	.01
Vascular dementia								
Unadjusted	1.19 (1.10-1.29)	<.001	1.12 (0.93-1.34)	.24	1.29 (1.22-1.37)	<.001	1.29 (1.20-1.39)	<.001
Adjusted^a^	1.44 (1.30-1.58)	<.001	1.31 (1.08-1.59)	.01	1.47 (1.36-1.59)	<.001	1.68 (1.52-1.86)	<.001
Depression								
Unadjusted	1.29 (1.20-1.38)	<.001	1.39 (1.19-1.62)	<.001	1.54 (1.48-1.61)	<.001	1.48 (1.40-1.58)	<.001
Adjusted^a^	1.61 (1.48-1.75)	<.001	1.68 (1.43-1.96)	<.001	1.95 (1.86-2.04)	<.001	2.21 (2.06-2.38)	<.001
Suicide								
Unadjusted	1.11 (0.94-1.32)	.23	0.87 (0.56-1.35)	.54	1.13 (1.01-1.26)	.04	1.41 (1.23-1.62)	<.001
Adjusted^a^	1.22 (0.99-1.49)	.06	0.98 (0.62-1.54)	.93	1.52 (1.34-1.72)	<.001	2.23 (1.87-2.66)	<.001

Abbreviations: 5-ARI, 5α-reductase inhibitor; HR, hazard ratio.

^a^ Model adjusted for year, β-blocker use, hypertension, obesity, type 2 diabetes, lipid disorders, and time exposed to 5-ARI or α-blocker. Models compare each drug against all individuals unexposed to any drug at time *t*.

For all drug types, compared with adjusted estimates, unadjusted estimates seemed to confound biasing toward the null (ie, negative confounding), underestimating the associations with the outcomes. Individuals taking finasteride and dutasteride showed an increased risk for all-cause dementia compared with unexposed individuals (finasteride: HR, 1.22 [95% CI, 1.17-1.28]; dutasteride: HR, 1.10 [95% CI, 1.01-1.20]) after adjustment (Table 2). Conversely, a reduced risk of dementia was observed for those who received α-blockers alone (HR, 0.71 [95% CI, 0.68-0.73]) or in combination with a 5-ARI (HR, 0.73 [95% CI, 0.70-0.77]). When comparing dementia risk between 5-ARIs, the risk associated with dutasteride was lower than the risk associated with finasteride (HR, 0.90 [95% CI, 0.81-0.99]; eTable 3 in Supplement 1). Regarding Alzheimer disease, all drugs showed an increased risk compared with unexposed individuals, with no differences between the drugs. For vascular dementia, all drugs presented an increased risk, but the risk was higher for those taking a combination of α-blockers and 5-ARIs compared with those taking α-blockers alone or finasteride (eTables 3 and 4 in Supplement 1). Compared with unexposed individuals, all drugs showed an increased risk for depression, and the risk appeared to be higher for exposures involving α-blockers alone (HR, 1.95 [95% CI, 1.86-2.04]) or in combination with a 5-ARI (HR, 2.21 [95% CI, 2.06-2.38]) (Table 2). Finasteride and dutasteride were not associated with suicide risk (finasteride: HR, 1.22 [95% CI, 0.99-1.49]; dutasteride: HR, 0.98 [95% CI, 0.62-1.54]), while exposures involving α-blockers, alone or combined with 5-ARIs, were associated with an increased risk for suicide (α-blockers alone: HR, 1.52 [95% CI, 1.34-1.72]; α-blockers combined with 5-ARIs: HR, 2.23 [95% CI, 1.87-2.66]).

When time exposed to each drug was examined, we observed that for those exposed to finasteride, compared with unexposed individuals, the risk for all-cause dementia was higher throughout the first 48 months and then became statistically nonsignificant beyond 4 years of exposure (Table 3). The association between dutasteride and all-cause dementia was inconsistent and statistically significant only during 13 to 48 months after starting treatment. The risk for all-cause dementia was lower for those receiving α-blocker treatment, alone or in combination with any 5-ARI, compared with unexposed individuals at any time during follow-up. For Alzheimer disease, compared with no treatment, treatment combining α-blockers and 5-ARIs was associated with lower risk, but all other treatments showed mostly statistically nonsignificant higher risks. In terms of vascular dementia, finasteride, α-blockers, and a combination of 5-ARIs and α-blockers were associated with higher risk compared with no treatment. We found an increased risk for vascular dementia among those receiving dutasteride treatment; however, only exposures between 13 and 48 months were associated with statistically significant risk. Concerning depression, all types of treatment were associated with higher risk compared with no treatment, and the magnitude of association was higher for treatments involving α-blockers than 5-ARIs. Across all medications, HRs appeared to decrease over time. Regarding suicide, no significant associations were found with 5-ARIs in any of the exposure periods, while treatment involving α-blockers was associated with a higher risk in the early periods.

**Table 3.** Results From the Time-Varying Cox Proportional Hazards Regression Model Stratified by Type of Drug Displaying the Association of Time Under Exposure With Outcomes^a^

Outcome and exposure period	Finasteride		Dutasteride		α-Blockers		α-Blockers and 5-ARI	
	HR (95% CI)	*P* value	HR (95% CI)	*P* value	HR (95% CI)	*P* value	HR (95% CI)	*P* value
All-cause dementia								
Unexposed	1 [Reference]	NA	1 [Reference]	NA	1 [Reference]	NA	1 [Reference]	NA
1-6 mo	1.20 (1.12-1.29)	<.001	1.04 (0.89-1.22)	.65	0.71 (0.67-0.75)	<.001	0.58 (0.54-0.62)	<.001
7-12 mo	1.24 (1.10-1.39)	<.001	0.97 (0.77-1.23)	.81	0.78 (0.71-0.85)	<.001	0.56 (0.50-0.63)	<.001
13-48 mo	1.13 (1.07-1.20)	<.001	1.17 (1.02-1.35)	.02	0.70 (0.66-0.76)	<.001	0.58 (0.54-0.62)	<.001
>49 mo	1.04 (0.97-1.12)	0.26	0.97 (0.82-1.15)	.75	0.74 (0.69-0.80)	<.001	0.55 (0.50-0.59)	<.001
Alzheimer disease								
Unexposed	1 [Reference]	NA	1 [Reference]	NA	1 [Reference]	NA	1 [Reference]	NA
1-6 mo	1.17 (1.02-1.34)	.03	1.25 (0.94-1.66)	.12	1.13 (1.04-1.24)	.01	0.82 (0.73-0.93)	<.001
7-12 mo	1.20 (0.95-1.52)	.12	1.13 (0.74-1.71)	.58	1.12 (0.96-1.31)	.15	0.83 (0.69-1.01)	.06
13-48 mo	1.12 (0.99-1.26)	.04	1.46 (1.14-1.86)	<.001	1.01 (0.89-1.15)	.84	0.83 (0.74-0.93)	<.001
>48 mo	0.98 (0.85-1.12)	.75	0.85 (0.60-1.21)	.38	1.12 (0.98-1.28)	.09	0.82 (0.71-0.94)	<.001
Vascular dementia								
Unexposed	1 [Reference]	NA	1 [Reference]	NA	1 [Reference]	NA	1 [Reference]	NA
1-6 mo	1.32 (1.12-1.54)	<.001	1.11 (0.77-1.59)	.59	1.32 (1.19-1.47)	<.001	1.30 (1.14-1.48)	<.001
7-12 mo	1.53 (1.18-1.99)	<.001	1.23 (0.75-2.01)	.40	1.56 (1.32-1.86)	<.001	1.46 (1.20-1.77)	<.001
13-48 mo	1.42 (1.25-1.61)	<.001	1.48 (1.10-1.99)	.01	1.62 (1.42-1.84)	<.001	1.27 (1.13-1.44)	<.001
>48 mo	1.13 (0.97-1.32)	.13	1.01 (0.68-1.51)	.95	1.71 (1.49-1.97)	<.001	1.21 (1.04-1.42)	.01
Depression								
Unexposed	1 [Reference]	NA	1 [Reference]	NA	1 [Reference]	NA	1 [Reference]	NA
1-6 mo	1.66 (1.47-1.87)	<.001	1.61 (1.23-2.11)	<.001	2.03 (1.92-2.15)	<.001	2.19 (2.02-2.38)	<.001
7-12 mo	1.61 (1.28-2.02)	<.001	1.15 (0.73-1.83)	.55	2.28 (2.06-2.54)	<.001	1.92 (1.67-2.22)	<.001
13-48 mo	1.43 (1.27-1.61)	<.001	1.84 (1.44-2.35)	<.001	1.77 (1.62-1.94)	<.001	1.96 (1.8-2.13)	<.001
>48 mo	1.35 (1.16-1.56)	<.001	1.31 (0.92-1.86)	.14	1.74 (1.55-1.96)	<.001	1.68 (1.47-1.91)	<.001
Suicide								
Unexposed	1 [Reference]	NA	1 [Reference]	NA	1 [Reference]	NA	1 [Reference]	NA
1-6 mo	1.16 (0.85-1.58)	.34	1.24 (0.62-2.48)	.55	1.49 (1.26-1.75)	<.001	1.33 (1.06-1.67)	.01
7-12 mo	1.16 (0.66-2.05)	.60	1.25 (0.47-3.34)	.65	1.96 (1.49-2.59)	<.001	1.20 (0.81-1.79)	.37
13-48 mo	1.07 (0.81-1.42)	.64	0.55 (0.21-1.46)	.23	1.43 (1.11-1.83)	.01	1.15 (0.90-1.46)	.26
>48 mo	0.97 (0.69-1.36)	.86	0.64 (0.24-1.71)	.37	1.34 (0.97-1.85)	.08	0.93 (0.65-1.34)	.70

Abbreviations: 5-ARI, 5α-reductase inhibitor; HR, hazard ratio; NA, not applicable.

^a^ Models adjusted for year, β-blockers, hypertension, obesity, diabetes, and lipid disorders and compare each drug against individuals unexposed to any drug at time *t.*

Results from sensitivity analyses defining the date of the dementia diagnosis as 3 years before the date of diagnosis and restricting analyses to those who started 5-ARI treatment at least 4 months after the start of the PDR are shown in the eAppendix and eTables 5, 6, 7, and 8 in Supplement 1. Results using a propensity score–matching approach (eTable 9 in Supplement 1) remained largely unchanged (eTables 10 and 11 in Supplement 1).

## Discussion

The aim of this register-based cohort study of men in Sweden aged 50 to 90 years was to assess the association of 5-ARI use with all-cause dementia, Alzheimer disease, vascular dementia, depression, and suicide. To our knowledge, this study has the largest sample size to date exploring these associations, including the associations with specific types of dementia. We have included a wide range of potential confounders and 14 years of follow-up.

The results from the main analyses showed a statistically significantly increased risk after adjustment for all-cause dementia, depression, Alzheimer disease, and vascular dementia for men receiving finasteride and dutasteride treatment compared with unexposed individuals, with a slightly higher magnitude of dementia risk for finasteride than dutasteride. In contrast, we found no evidence of an increased risk for suicide among individuals taking 5-ARIs. We also found a decreased risk for all-cause dementia among those taking α-blockers. This result is consistent with a recent population-based study in which a decreased risk for dementia was observed among those using BPH medications.^26^ Furthermore, when we examined time exposed to 5-ARIs, the associations with all-cause dementia, Alzheimer disease, and vascular dementia decreased over time and ultimately became nonstatistically significant, whereas the risk for depression seemed to be stable over time, with a negligible decrease with exposures longer than 3 years. These findings are in agreement with prior research insofar as both finasteride and dutasteride seem to be associated with a higher overall risk for dementia and depression and seem to have no significant association with suicide.^9,15^ Moreover, associations were attenuated over time and became no longer statistically significant with longer exposures. It is possible that the initially higher magnitude of risk reflects the risk of the individuals most susceptible to dementia and that the decrease in risk over time was due to the depletion of such susceptible individuals (ie, those who were more prone to develop dementia might have developed it first, thereby “depleting” susceptible individuals).^27^ However, the decreasing trend mirrors previous research^9,15^ and may suggest the presence of surveillance bias, by which the increased risk for dementia might be due to increased detection of dementia among patients with BPH. Finally, when restricting 5-ARI users to those who started treatment at least 4 months after the inception of the PDR to include only new 5-ARI users to accurately account for time exposed to 5-ARIs, associations of 5-ARIs with dementia and Alzheimer disease were no longer significant. We could see only sporadic associations of 5-ARIs with depression and vascular dementia. Therefore, both finasteride and dutasteride do not seem to be associated with an increased risk for all-cause dementia, Alzheimer disease, vascular dementia, or suicide. However, individuals receiving 5-ARI treatment consistently showed an increased risk for depression compared with unexposed individuals across various analyses. The increased risk for vascular dementia among men receiving α-blockers alone or combined with 5-ARIs was unexpected. This finding may be explained by the fact that α-blockers are prescribed not only for BPH but also to treat high blood pressure, a risk factor for vascular dementia.^28^

The lack of an association between 5-ARIs and dementia is broadly consistent with the results of other studies in this area, in which a lack of and association between androgens (ie, DHT and testosterone) and cognition^29,30^ was seen; however, positive associations can be found in the literature.^4,31^ The underlying mechanism by which low DHT is associated with poor cognitive function is unclear. First, it is plausible that given the significant expression of *SRD5A1* (OMIM 184753) and *SRD5A3* (OMIM 611715) in androgen-responsive tissues of the brain^32,33^ and that 5-ARIs inhibit steroid 5α-reductases (finasteride specifically inhibits the activity of *SRD5A2* (OMIM 607306) and *SRD5A3*, and dutasteride inhibits *SRD5A1* and *SRD5A3*), there might be a putative, tissue-specific association of DHT inhibition with both cognitive function and mood. Second, neuron death is a pathological feature of Alzheimer disease, and prior evidence has demonstrated that androgens are of great importance in axonal regeneration and neuron growth.^34^ Last, a higher risk for dementia was also observed among individuals undergoing androgen deprivation therapy.^6^ However, research on DHT and dementia (even more on 5-ARIs) is scarce, with contradictory findings, and thus more research is clearly warranted.

The statistically significant associations between 5-ARIs and depression are consistent with prior evidence^8,12,13^ and seem to emphasize the role of 5-AR in mood regulation. This association could be ascribed to not only the decrease in DHT formation but also to serum levels of a broad set of 5α-reduced steroids that are considered neuroactive steroids that act on γ-aminobutyric acid type A and *N*-methyl-D-aspartate receptors.^18,35^ Individuals with depression showed both decreased 5-AR activity and 5α-reduced steroid concentrations^36^ that increased with antidepressant treatment.^37^ Furthermore, this hypothesis might be strengthened by the fact that the antidepressant effect seems to be inhibited when antidepressants are combined with finasteride, suggesting that finasteride might be associated with a reduction in neuroactive steroids.^38^

A possible explanation for the positive associations of 5-ARIs with all-cause dementia and vascular dementia that we observed in some of our analyses is that these associations are confounded. In our study population, men treated with 5-ARIs had a greater number of comorbid diseases, which inherently increases the likelihood of dementia detection, leading to surveillance bias. By comparing 5-ARI use with α-blocker treatment, which is also used for treating BPH and previously shown not to be associated with dementia, we attempted to detect possible surveillance bias. In secondary analyses, we observed a constant, decreased risk over time for all-cause dementia and an increased risk for vascular dementia in men taking α-blockers; however, the risk over time for those taking 5-ARIs decreased over time, further supporting the possibility of surveillance bias. Therefore, surveillance bias and residual confounding by indication cannot be ruled out as a source of bias in the estimates for dementia. The association between 5-ARIs and depression was constant and thus unlikely to be solely due to surveillance bias.

### Strengths and Limitations

This study has some strengths, including the use of national registers, resulting in an unselected, large population with validated clinical diagnoses.^39^ Second, we identified individuals receiving 5-ARI treatment as well as other treatments that were identified using the PDR, which records all dispensed medications in Sweden, including those for conditions that were treated at primary care. Third, the longitudinal nature of the data without any missing data on follow-up allowed us to closely monitor each individual for more than 13 years of observation.

The results should also be considered in light of some potential limitations. Misclassification of disease is a major issue in register-based registries. Previous research showed a 55% sensitivity and 98% specificity for dementia in Swedish registries.^25^ We attempted to minimize this limitation by including drugs used to treat dementia to identify more patients with dementia who were not included in the PDR (which covers >99% of individuals). However, information on prescribed drugs was available from 2005 onward. Nonetheless, misclassification is plausible and could produce a bias toward the null, assuming that there is a true positive association of 5-ARIs with our outcomes. Likewise, we should take into account that some individuals do not seek treatment, and therefore we were unable to identify them, which would potentially result in an underestimation or overestimation of 5-ARI–associated depression. Similarly, measurements of hypertension, obesity, type 2 diabetes, and lipid disorders likely contain some misclassification, which could potentially have affected our estimates. Some estimates may have been underpowered and thus need to be interpreted with caution. For instance, the incidence of suicide is low among the elderly population, and thus the nonstatistically significant association with 5-ARIs may in fact be partly associated with a lack of statistical power. Similarly, when restricting 5-ARI users to those who started treatment at least 4 months after the start of the PDR, the statistical power for both 5-ARIs in depression and suicide analyses appeared low. Another noteworthy limitation is that by using registry data, it was not possible to assess whether an individual was taking the medication as directed or to obtain information regarding the prescribed dose. Furthermore, owing to statistical power constraints, when restricting analyses to those who started 5-ARI treatment 4 months after the beginning of the PDR, estimates became more imprecise, particularly for those using dutasteride. Further work with longer follow-up times is required to shed more light on this association and to determine whether 5-ARIs are associated with all types of dementia or a specific type.

## Conclusions

The findings of this cohort study suggest that, given the decreasing magnitude of the association over time, 5-ARI medications—finasteride and dutasteride—do not seem to be associated with all-cause dementia, Alzheimer disease, vascular dementia, or suicide. However, individuals receiving 5-ARI treatment may be at increased risk for depression.

## Notes

Supplement 1. **eTable 1.** *ICD* and *ATC* Codes Used to Define Outcomes and Covariates **eTable 2.** Person-years, Number of Events, and Incidence Rate With 95% CI for Each Type of Drug and Outcome **eTable 3.** Results From the Time-Varying Cox Model (HR and 95% CI) Displaying the Association Between Each Type of Drug With All-Cause Dementia, Depression, Suicide, Alzheimer’s Disease, and Vascular Dementia Compared to Finasteride Users **eTable 4.** Results From the Time-Varying Cox Model (HR and 95% CI) Displaying the Association Between Each Type of Drug With All-Cause Dementia, Depression, Suicide, Alzheimer’s Disease, and Vascular Dementia Compared With Individuals Taking Alpha Blockers **eTable 5.** Results From the Time-Varying Cox Model (HR and 95% CI) for All-Cause Dementia Defined as Three Years Before the Date of Diagnosis **eTable 6.** Results From the Time-Varying Cox Model (HR and 95% CI) Stratified by Type of Drug for All-Cause Dementia Defined as Three Years Before the Date of Diagnosis **eTable 7.** Results From the Time-Varying Cox Model (HR and 95% CI) With 5ARI Users Restricted to Those Who Started Treatment at Least 4 Months After July 2005 **eTable 8.** Results From the Time-Varying Cox Model (HR and 95% CI) With 5ARI Users Restricted to Those Who Started Treatment 4 Months After July 2005 **eTable 9.** Descriptive Statistics of the Cohort Stratified by Drug Exposure During Follow-up **eTable 10.** Results From the Cox Model (HR and 95% CI) Comparing 5ARI Users and Alpha Blockers to Matched Controls **eTable 11.** Results From the Time-Varying Cox Model (HR and 95% CI) Stratified by Type of Drug Displaying the Association of Time Under Exposure With Dementia, Alzheimer’s Disease, Vascular Dementia, Depression, and Suicide
eMethods.

eAppendix.

Supplement 2.
Data Sharing Statement
