# Supplementary material for: Association of 5α-Reductase Inhibitors With Dementia, Depression, and Suicide
Source: JAMA Netw Open. 2022 Dec 22;5(12):e2248135. doi: 10.1001/jamanetworkopen.2022.48135 (PMC9857015; doi:10.1001/jamanetworkopen.2022.48135)
Supplement: Supplement 1. — eTable 1. ICD and ATC Codes Used to Define Outcomes and Covariates eTable 2. Person-years, Number of Events, and Incidence Rate With 95% CI for Each Type of Drug and Outcome eTable 3. Results From the Time-Varying Cox Model (HR and 95% CI) Displaying the Association Between Each Type of Drug With All-Cause Dementia, Depression, Suicide, Alzheimer’s Disease, and Vascular Dementia Compared to Finasteride Users eTable 4. Results From the Time-Varying Cox Model (HR and 95% CI) Displaying the Association Between Each Type of Drug With All-Cause Dementia, Depression, Suicide, Alzheimer’s Disease, and Vascular Dementia Compared With Individuals Taking Alpha Blockers eTable 5. Results From the Time-Varying Cox Model (HR and 95% CI) for All-Cause Dementia Defined as Three Years Before the Date of Diagnosis eTable 6. Results From the Time-Varying Cox Model (HR and 95% CI) Stratified by Type of Drug for All-Cause Dementia Defined as Three Years Before the Date of Diagnosis eTable 7. Results From the Time-Varying Cox Model (HR and 95% CI) With 5ARI Users Restricted to Those Who Started Treatment at Least 4 Months After July 2005 eTable 8. Results From the Time-Varying Cox Model (HR and 95% CI) With 5ARI Users Restricted to Those Who Started Treatment 4 Months After July 2005 eTable 9. Descriptive Statistics of the Cohort Stratified by Drug Exposure During Follow-up eTable 10. Results From the Cox Model (HR and 95% CI) Comparing 5ARI Users and Alpha Blockers to Matched Controls eTable 11. Results From the Time-Varying Cox Model (HR and 95% CI) Stratified by Type of Drug Displaying the Association of Time Under Exposure With Dementia, Alzheimer’s Disease, Vascular Dementia, Depression, and Suicide eMethods. eAppendix. [file jamanetwopen-e2248135-s001.pdf]

Supplementary Online Content

Garcia-Argibay M, Hiyoshi A, Fall K, Montgomery S. Association of 5α-reductase inhibitors with dementia, depression, and suicide. *JAMA Netw Open.* 2022;5(12):e2248135. doi:10.1001/jamanetworkopen.2022.48135

eTable 1. *ICD* and *ATC* Codes Used to Define Outcomes and Covariates

eTable 2. Person-years, Number of Events, and Incidence Rate With 95% CI for Each Type of Drug and Outcome

eTable 3. Results From the Time-Varying Cox Model (HR and 95% CI) Displaying the Association Between Each Type of Drug With All-Cause Dementia, Depression, Suicide, Alzheimer's Disease, and Vascular Dementia Compared to Finasteride Users

eTable 4. Results From the Time-Varying Cox Model (HR and 95% CI) Displaying the Association Between Each Type of Drug With All-Cause Dementia, Depression, Suicide, Alzheimer's Disease, and Vascular Dementia Compared With Individuals Taking Alpha Blockers

eTable 5. Results From the Time-Varying Cox Model (HR and 95% CI) for All-Cause Dementia Defined as Three Years Before the Date of Diagnosis

eTable 6. Results From the Time-Varying Cox Model (HR and 95% CI) Stratified by Type of Drug for All-Cause Dementia Defined as Three Years Before the Date of Diagnosis

eTable 7. Results From the Time-Varying Cox Model (HR and 95% CI) With 5ARI Users Restricted to Those Who Started Treatment at Least 4 Months After July 2005

eTable 8. Results From the Time-Varying Cox Model (HR and 95% CI) With 5ARI Users Restricted to Those Who Started Treatment 4 Months After July 2005

eTable 9. Descriptive Statistics of the Cohort Stratified by Drug Exposure During Follow-up

eTable 10. Results From the Cox Model (HR and 95% CI) Comparing 5ARI Users and Alpha Blockers to Matched Controls

eTable 11. Results From the Time-Varying Cox Model (HR and 95% CI) Stratified by Type of Drug Displaying the Association of Time Under Exposure With Dementia, Alzheimer's Disease, Vascular Dementia, Depression, and Suicide

eMethods.

eAppendix.

This supplementary material has been provided by the authors to give readers additional information about their work.

© 2022 Garcia-Argibay M et al. *JAMA Network Open.*

eTable 1. *ICD* and *ATC* Codes Used to Define Outcomes and Covariates

	ICD-10	*ICD-9*	*ICD-8*	*ATC*
Outcomes				
Dementia	F00-F01, F02.3, F03, F05.1, F09, G30, G31.1, or R54	290A, 290B, 290E, 290W, 290X, 294B, 331A-C, 331X	290, 293.0-293.1	N06DA01, N06DA03, N06DA04, N06DX01, N06DA02, N06DA52
Alzheimer's disease	F00, F03, G30	290A/B/X, 331A	290	NA
Vascular dementia	F01	290E	293.0-293.1	NA
Depression	F32-F34	296B, 300E	296.2, 296.9, 298.0, 300.4	NA
Suicide	X60-X84	950–959		NA
Covariates				
Finasteride	NA	NA	NA	G04CB01
Dutasteride	NA	NA	NA	G04CB02
Alpha blockers	NA	NA	NA	G04CA01-G04CA04, C02CA04
Beta blockers	NA	NA	NA	C07A
Obesity	E66	278A-B	277	NA
Eating disorders	F50.0-F50.3, F50.9	307B, 307F		NA
Hypertension	I10-I15, or I67.4	401-405	400-404	NA
Diabetes	E10-14	250	250	NA
Lipid disorders	E78.0-E78.9	272.0, 272.1		NA

Note. NA = Not applicable

© 2022 Garcia-Argibay M et al. *JAMA Network Open.*

eTable 2. Person-years, Number of Events, and Incidence Rate With 95% CI for Each Type of Drug and Outcome

Outcome	Unexposed	Finasteride	Dutasteride	Alpha blockers	Alpha blockers and 5ARI
**All-cause dementia**					
Person-years	18371610.95	449042.9	83550.96	1132501.64	571787.93
Events	40356	3561	572	4069	2847
Incidence rate	21.97 (21.75-22.18)	79.3 (76.72-81.95)	68.46 (62.97-74.31)	35.93 (34.83-37.05)	49.79 (47.98-51.65)
**Alzheimer's disease**					
Person-years	18475935.69	458287.79	84936.78	1143439.46	579445.92
Events	11055	892	170	1447	962
Incidence rate	5.98 (5.87-6.1)	19.46 (18.21-20.78)	20.01 (17.12-23.26)	12.65 (12.01-13.32)	16.6 (15.57-17.69)
**Vascular dementia**					
Person-years	18486892.01	458547.62	85055.06	1143982.11	579714.46
Events	7454	689	115	1173	819
Incidence rate	4.03 (3.94-4.12)	15.03 (13.92-16.19)	13.52 (11.16-16.23)	10.25 (9.68-10.86)	14.13 (13.18-15.13)
**Depression**					
Person-years	18073479.09	450009.96	83463.55	1108197.98	565440.62
Events	33184	846	167	2595	1202
Incidence rate	18.36 (18.16-18.56)	18.8 (17.55-20.11)	20.01 (17.09-23.28)	23.42 (22.52-24.34)	21.26 (20.07-22.49)
**Suicide**					
Person-years	18516038.26	461279	85488.88	1148502.11	582827.84
Events	4793	141	20	331	216
Incidence rate	2.59	3.06	2.34	2.88	3.71

© 2022 Garcia-Argibay M et al. *JAMA Network Open.*

	(2.52-2.66)	(2.57-3.6)	(1.43-3.61)	(2.58-3.21)	(3.23-4.23)
**Dementia***					
Person-years	18258955.09	440552.34	82037.07	1122733.89	565304.81
Events	34210	2190	435	2569	1568
Incidence rate	18.74 (18.54-18.94)	49.71 (47.65-51.84)	53.02 (48.16-58.25)	22.88 (22.01-23.78)	27.74 (26.38-29.14)

Note. Incidence rates calculated per 10000 person-years. *Dementia onset was set as 3 years before the diagnosis.

© 2022 Garcia-Argibay M et al. *JAMA Network Open.*

eTable 3. Results From the Time-Varying Cox Model (HR and 95% CI) Displaying the Association Between Each Type of Drug With All-Cause Dementia, Depression, Suicide, Alzheimer's Disease, and Vascular Dementia Compared to Finasteride Users

		**Unexposed**			**Dutasteride**			**Alpha blockers**			**5ARI and alpha blockers**		
	**Model**	**HR**		**P**	**HR**		**P**	**HR**		**P**	**HR**		**P**
All-cause dementia	Unadjusted	0.91	(0.88-0.94)	<.001	0.91	(0.82-0.99)	.04	0.75	(0.70-0.80)	<.001	0.74	(0.69-0.79)	<.001
	Adjusted[a]	0.82	(0.77-0.87)	<.001	0.90	(0.81-0.99)	.02	0.58	(0.53-0.63)	<.001	0.60	(0.55-0.65)	<.001
Alzheimer's disease	Unadjusted	0.98	(0.91-1.05)	.87	1.09	(0.93-1.25)	.60	1.06	(0.98-1.14)	.60	1.00	(0.91-1.09)	.92
	Adjusted[a]	0.83	(0.74-0.92)	<.001	1.06	(0.90-1.22)	.48	0.92	(0.82-1.02)	.16	0.95	(0.86-1.04)	.36
Vascular dementia	Unadjusted	0.84	(0.76-0.92)	<.001	0.94	(0.74-1.14)	.53	1.09	(0.99-1.18)	.15	1.09	(0.99-1.19)	.15
	Adjusted[a]	0.70	(0.60-0.80)	<.001	0.91	(0.71-1.11)	.48	1.03	(0.92-1.14)	.65	1.17	(1.06-1.28)	.006
Depression	Unadjusted	0.78	(0.71-0.85)	<.001	1.08	(0.91-1.25)	.37	1.20	(1.12-1.28)	<.001	1.15	(1.06-1.24)	<.001
	Adjusted[a]	0.62	(0.54-0.70)	<.001	1.04	(0.87-1.21)	.63	1.21	(1.12-1.30)	<.001	1.38	(1.29-1.47)	<.001
Suicide	Unadjusted	0.90	(0.73-1.07)	.41	0.78	(0.31-1.25)	.41	1.01	(0.81-1.21)	.89	1.27	(1.06-1.48)	.11
	Adjusted[a]	0.82	(0.62-1.02)	.078	0.81	(0.34-1.28)	.37	1.25	(1.02-1.48)	.08	1.83	(1.61-2.05)	<.001

Note. [a]Model adjusted for beta blockers, hypertension, obesity, diabetes, lipid disorders, and cumulative 5ARI/alpha blocker exposure. Models compare each drug against individuals unexposed to any of the drugs at time *t*.

© 2022 Garcia-Argibay M et al. *JAMA Network Open.*

eTable 4. Results From the Time-Varying Cox Model (HR and 95% CI) Displaying the Association Between Each Type of Drug With All-Cause Dementia, Depression, Suicide, Alzheimer's Disease, and Vascular Dementia Compared With Individuals Taking Alpha Blockers

		**Unexposed**			**Finasteride**			**Dutasteride**			**5ARI and alpha blockers**		
	**Model**	**HR**		**P**	**HR**		**P**	**HR**		**P**	**HR**		**P**
All-cause dementia	Unadjusted	1.22	(1.19-1.25)	<.001	1.34	(1.29-1.39)	<.001	1.22	(1.13-1.31)	<.001	0.99	(0.94-1.04)	.67
	Adjusted[a]	1.42	(1.38-1.46)	<.001	1.73	(1.68-1.78)	.15	1.56	(1.47-1.65)	<.001	1.04	(0.99-1.09)	<.001
Alzheimer's disease	Unadjusted	0.93	(0.87-0.99)	.02	0.94	(0.86-1.02)	.23	1.03	(0.87-1.19)	.75	0.95	(0.87-1.03)	.23
	Adjusted[a]	0.91	(0.84-0.98)	.02	1.09	(0.99-1.19)	.46	1.16	(0.99-1.33)	.11	1.04	(0.95-1.13)	.11
Vascular dementia	Unadjusted	0.77	(0.71-0.83)	<.001	0.92	(0.83-1.01)	.16	0.86	(0.67-1.05)	.18	1.00	(0.91-1.09)	.97
	Adjusted[a]	0.68	(0.6-0.76)	<.001	0.98	(0.87-1.09)	.02	0.89	(0.69-1.09)	.33	1.14	(1.04-1.24)	.65
Depression	Unadjusted	0.65	(0.61-0.69)	<.001	0.83	(0.75-0.91)	<.001	0.90	(0.74-1.06)	.25	0.96	(0.89-1.03)	.26
	Adjusted[a]	0.51	(0.46-0.56)	<.001	0.82	(0.73-0.91)	.002	0.86	(0.70-1.02)	.07	1.13	(1.05-1.21)	<.001
Suicide	Unadjusted	0.89	(0.78-1.00)	.07	0.99	(0.79-1.19)	.89	0.77	(0.32-1.22)	.35	1.25	(1.08-1.42)	.04
	Adjusted[a]	0.66	(0.53-0.79)	<.001	0.80	(0.57-1.03)	<.001	0.65	(0.18-1.12)	.06	1.47	(1.28-1.66)	.06

Note. [a]Model adjusted for beta blockers, hypertension, obesity, diabetes, lipid disorders, and cumulative 5ARI/alpha blocker exposure. Models compare each drug against individuals unexposed to any of the drugs at time *t*.

© 2022 Garcia-Argibay M et al. *JAMA Network Open.*

eTable 5. Results From the Time-Varying Cox Model (HR and 95% CI) for All-Cause Dementia Defined as Three Years Before the Date of Diagnosis

**Condition**	**Unadjusted**	**P**	**Adjusted**[a]	**P**
Unexposed	Ref		Ref	
Finasteride	0.99 (0.95-1.03)	.68	1.17 (1.11-1.24)	<0.01
Dutasteride	1.08 (0.98-1.18)	.13	1.25 (1.13-1.38)	<0.01
Alpha blockers	0.66 (0.63-0.69)	<0.01	0.96 (0.92-1.01)	.14
5ARI and alpha blockers	0.6 (0.57-0.64)	<0.01	1.07 (0.99-1.14)	.05

Note. [a]Model adjusted for year, beta blockers, hypertension, obesity, diabetes, lipid disorders, and cumulative 5ARI/alpha blocker exposure. Models compare each drug against individuals unexposed to any of the drugs at time *t*.

© 2022 Garcia-Argibay M et al. *JAMA Network Open.*

eTable 6. Results From the Time-Varying Cox Model (HR and 95% CI) Stratified by Type of Drug for All-Cause Dementia Defined as Three Years Before the Date of Diagnosis

Exposure	Finasteride	P	Dutasteride	P	Alpha blockers	P	5ARI and alpha blockers	P
Unexposed	-		-		-		-	
1 to 6 months	1.53 (1.41-1.66)	<.001	1.46 (1.24-1.73)	<.001	0.93 (0.87-1.00)	.04	1.09 (1.01-1.18)	.03
7 to 12 months	1.54 (1.33-1.79)	<.001	1.42 (1.10-1.83)	.01	1.00 (0.89-1.12)	.96	1.07 (0.94-1.21)	.32
13 to 48 months	1.44 (1.34-1.55)	<.001	1.72 (1.47-2.01)	<.001	1.11 (1.02-1.21)	.02	1.12 (1.04-1.21)	<.001
> 48 months	1.56 (1.42-1.71)	<.001	1.39 (1.10-1.75)	.01	0.85 (0.76-0.95)	<.001	0.86 (0.77-0.97)	.01

Note. Models adjusted for year, beta blockers, hypertension, obesity, diabetes, lipid disorders, and cumulative 5ARI/alpha blocker exposure. Models compare each drug separately against individuals unexposed to the given drug at time *t*.

© 2022 Garcia-Argibay M et al. *JAMA Network Open.*

eTable 7. Results From the Time-Varying Cox Model (HR and 95% CI) With 5ARI Users Restricted to Those Who Started Treatment at Least 4 Months After July 2005

		Finasteride		Dutasteride		Alpha blockers		5ARI and alpha blockers	
	**Model**	**HR**	**P**	**HR**	**P**	**HR**	**P**	**HR**	**P**
All-cause dementia	Unadjusted	0.98 (0.92-1.04)	.49	1.13 (0.94-1.36)	.19	0.83 (0.80-0.86)	<.001	0.91 (0.80-1.04)	.17
	Adjusted[a]	1.07 (0.97-1.17)	.19	1.23 (1.01-1.49)	.04	0.74 (0.71-0.77)	<.001	0.87 (0.75-1.02)	.09
Alzheimer's disease	Unadjusted	0.94 (0.83-1.07)	.35	1.10 (0.77-1.57)	.60	1.11 (1.05-1.17)	<.001	1.13 (0.91-1.41)	.27
	Adjusted[a]	0.96 (0.80-1.17)	.71	1.13 (0.77-1.65)	.54	1.15 (1.06-1.24)	<.001	1.13 (0.85-1.50)	.39
Vascular dementia	Unadjusted	1.19 (1.03-1.37)	.02	0.99 (0.62-1.57)	.96	1.31 (1.23-1.40)	<.001	1.51 (1.19-1.91)	<.001
	Adjusted[a]	1.29 (1.05-1.59)	.02	1.05 (0.65-1.70)	.83	1.51 (1.39-1.64)	<.001	1.79 (1.32-2.43)	<.001
Depression	Unadjusted	1.21 (1.04-1.40)	.01	1.48 (1.01-2.15)	.04	1.55 (1.49-1.62)	<.001	1.79 (1.42-2.25)	<.001
	Adjusted[a]	1.29 (1.05-1.59)	.02	1.56 (1.05-2.33)	.03	1.99 (1.90-2.09)	<.001	2.34 (1.75-3.13)	<.001
Suicide	Unadjusted	1.07 (0.77-1.50)	.69	1.55 (0.69-3.44)	.29	1.21 (1.08-1.36)	<.001	1.55 (0.92-2.63)	.10
	Adjusted[a]	1.48 (0.93-2.35)	.10	2.06 (0.88-4.78)	.09	1.67 (1.47-1.90)	<.001	3.18 (1.68-6.03)	<.001

Note. [a]Model adjusted for year, beta blockers, hypertension, obesity, diabetes, lipid disorders, and cumulative 5ARI/alpha blocker exposure. Models compare each drug against individuals unexposed to any of the drugs at time *t*.

© 2022 Garcia-Argibay M et al. *JAMA Network Open.*

eTable 8. Results From the Time-Varying Cox Model (HR and 95% CI) With 5ARI Users Restricted to Those Who Started Treatment 4 Months After July 2005

		Finasteride		Dutasteride		Alpha blockers		Alpha blockers and 5ARI	
Outcome	Period	HR	P	HR	P	HR	P	HR	P
Dementia	Unexposed	REF		REF		REF		REF	
	1 to 6 months	1.07 (0.92-1.25)	.40	1.26 (0.88-1.81)	.20	0.71 (0.67-0.75)	<.001	0.54 (0.38-0.76)	<.001
	7 to 12 months	1.05 (0.80-1.37)	.75	1.11 (0.64-1.91)	.71	0.80 (0.72-0.87)	<.001	0.66 (0.41-1.06)	.09
	13 to 48 months	0.98 (0.88-1.08)	.65	1.41 (1.01-1.89)	.02	0.74 (0.69-0.08)	<.001	0.53 (0.44-0.65)	<.001
	>48 months	0.94 (0.85-1.05)	.26	0.80 (0.55-1.17)	.26	0.76 (0.70-0.82)	<.001	0.58 (0.49-0.68)	<.001
Alzheimer's disease	Unexposed	REF		REF		REF		REF	
	1 to 6 months	0.75 (0.52-1.09)	.13	1.28 (0.64-2.56)	.49	1.16 (1.06-1.27)	<.001	0.48 (0.23-1.01)	.05
	7 to 12 months	0.86 (0.48-1.56)	.62	1.63 (0.68-3.93)	.27	1.19 (1.02-1.40)	.03	0.81 (0.34-1.95)	.64
	13 to 48 months	1.02 (0.83-1.25)	.85	1.54 (0.89-2.65)	.12	1.08 (0.95-1.24)	.22	0.71 (0.50-1.02)	.06
	>48 months	1.01 (0.83-1.23)	.92	0.46 (0.17-1.21)	.12	1.17 (1.02-1.33)	.03	0.85 (0.64-1.13)	.26
Vascular dementia	Unexposed	REF		REF		REF		REF	
	1 to 6 months	1.12 (0.77-1.63)	.54	0.47 (0.12-1.87)	.28	1.35 (1.21-1.50)	<.001	1.25 (0.69-2.26)	.46
	7 to 12 months	1.35 (0.75-2.43)	.32	1.49 (0.48-4.63)	.49	1.55 (1.30-1.86)	<.001	1.39 (0.58-3.35)	.46
	13 to 48 months	1.39 (1.12-1.73)	<.001	1.23 (0.59-2.59)	.58	1.75 (1.53-2.00)	<.001	0.85 (0.55-1.03)	.45
	> 48 months	1.12 (0.89-1.41)	.32	1.03 (0.46-2.31)	.93	1.72 (1.49-1.99)	<.001	1.41 (1.04-1.9)	.03
Depression	Unexposed	REF		REF		REF		REF	
	1 to 6 months	1.13 (0.78-1.62)	.52	1.20 (0.54-2.68)	.65	2.05 (1.93-2.18)	<.001	0.69 (0.33-1.46)	.34
	7 to 12 months	1.36 (0.75-2.45)	.31	2.51 (1.04-6.03)	.04	2.32 (2.07-2.60)	<.001	2.85 (1.58-5.14)	<.001
	13 to 48 months	1.41 (1.13-1.77)	<.001	2.12 (1.20-3.74)	.01	1.79 (1.62-1.98)	<.001	1.90 (1.40-2.57)	<.001
	> 48 months	1.16 (0.91-1.49)	.23	0.81 (0.30-2.15)	.67	1.74 (1.53-1.97)	<.001	2.06 (1.54-2.75)	<.001
Suicide	Unexposed	REF		REF		REF		REF	
	1 to 6 months	1.07 (0.45-2.59)	.87	2.22 (0.55-8.87)	.26	1.62 (1.37-1.90)	<.001	1.09 (0.27-4.37)	.9

© 2022 Garcia-Argibay M et al. *JAMA Network Open.*

	7 to 12 months	2.54 (0.95-6.78)	.06	2.69 (0.38-19.11)	.32	2.19 (1.66-2.90)	<.001	2.65 (0.66-10.62)	.17
	13 to 48 months	1.36 (0.82-2.27)	.23	1.71 (0.43-6.83)	.45	1.63 (1.27-2.10)	<.001	0.70 (0.22-2.170)	.53
	> 48 months	0.77 (0.42-1.39)	.38	0.79 (0.11-5.62)	.81	1.47 (1.06-2.03)	.02	1.42 (0.67-2.98)	.36

Note. Models adjusted for year, beta blockers, hypertension, obesity, diabetes, and lipid disorders. Models compare each drug separately against nonexposed individuals at time *t*.

© 2022 Garcia-Argibay M et al. *JAMA Network Open*.

eTable 9. Descriptive Statistics of the Cohort Stratified by Drug Exposure During Follow-up

**Characteristic**	**Matched controls (N = 1,019,704)**	**Finasteride (N = 69,967)**	**Dutasteride (N = 8,544)**	**Alpha (N = 181,942)**
Birth year (IQR)	1,945 (1,935, 1,952)	1,936 (1,928, 1,944)	1,933 (1,926, 1,940)	1,945 (1,936, 1,952)
Age at death, years (IQR)	80 (72, 86)	86 (81, 90)	85 (80, 89)	82 (75, 88)
Covariates, n (%)				
Beta blockers	405,064 (40%)	35,866 (51%)	4,622 (54%)	0 (0%)
Type 2 diabetes	84,035 (8.2%)	5,768 (8.2%)	741 (8.7%)	12,874 (7.1%)
Hypertension	93,933 (9.2%)	7,544 (11%)	979 (11%)	15,523 (8.5%)
Obesity	8,308 (0.8%)	421 (0.6%)	41 (0.5%)	1,915 (1.1%)
Lipid disorder	8,873 (0.9%)	654 (0.9%)	76 (0.9%)	1,973 (1.1%)
Eating disorder	141 (<0.1%)	15 (<0.1%)	1 (<0.1%)	30 (<0.1%)
Outcomes, n (%)				
Dementia	36,828 (3.6%)	4,376 (6.3%)	606 (7.1%)	4,851 (2.7%)
Alzheimer's disease	9,732 (1.0%)	1,076 (1.5%)	177 (2.1%)	1,666 (0.9%)
Vascular dementia	6,796 (0.7%)	847 (1.2%)	128 (1.5%)	1,311 (0.7%)
Depression	31,995 (3.1%)	2,292 (3.3%)	285 (3.3%)	7,741 (4.3%)
Completed suicide	3,290 (0.3%)	141 (0.2%)	20 (0.2%)	313 (0.2%)

© 2022 Garcia-Argibay M et al. *JAMA Network Open.*

eTable 10. Results From the Cox Model (HR and 95% CI) Comparing 5ARI Users and Alpha Blockers to Matched Controls

Outcome	Finasteride	p	Dutasteride	p	Alpha blockers	p
All-cause dementia	1.15 (1.10-1.19)	<.001	1.03 (0.92-1.18)	.57	0.68 (0.66-0.71)	<.001
Alzheimer's disease	1.02 (0.94-1.10)	.68	1.09 (0.88-1.35)	.44	0.86 (0.81-0.92)	<.001
Vascular dementia	1.14 (1.04-1.25)	.004	1.02 (0.79-1.32)	.86	0.96 (0.89-1.03)	.26
Depression	1.15 (1.07-1.23)	<.001	1.54 (1.27-1.88)	<.001	1.42 (1.37-1.47)	<.001
Suicide	0.59 (0.48-0.73)	<.001	0.60 (0.32-1.13)	.11	0.49 (0.43-0.56)	<.001

© 2022 Garcia-Argibay M et al. *JAMA Network Open.*

eTable 11. Results From the Time-Varying Cox Model (HR and 95% CI) Stratified by Type of Drug Displaying the Association of Time Under Exposure With Dementia, Alzheimer's Disease, Vascular Dementia, Depression, and Suicide

Outcome	Period	Finasteride	P	Dutasteride	P	Alpha blockers	P
All-cause dementia	Unexposed	REF		REF		REF	
	1 to 6 months	1.67 (1.51-1.84)	<.001	1.14 (0.90-1.46)	.28	0.82 (0.77-0.88)	<.001
	7 to 12 months	1.62 (1.38-1.90)	<.001	1.25 (0.90-1.74)	.18	0.97 (0.86-1.11)	.68
	13 to 48 months	1.35 (1.25-1.45)	<.001	1.19 (0.97-1.46)	.09	0.89 (0.80-0.98)	.01
	> 49 months	0.99 (0.91-1.08)	.79	0.94 (0.73-1.20)	.66	0.85 (0.76-0.95)	.005
Alzheimer's disease	Unexposed	REF		REF		REF	
	1 to 6 months	1.55 (1.28-1.89)	<.001	1.55 (1.01-2.36)	.04	1.19 (1.06-1.34)	.004
	7 to 12 months	1.29 (0.93-1.79)	.12	1.17 (0.64-2.13)	.61	1.40 (1.13-1.74)	.002
	13 to 48 months	1.15 (0.99-1.33)	.05	1.34 (0.92-1.96)	.12	1.06 (0.89-1.26)	.53
	> 48 months	0.91 (0.77-1.09)	.31	0.60 (0.35-1.02)	.06	1.19 (0.98-1.45)	.08
Vascular dementia	Unexposed	REF		REF		REF	
	1 to 6 months	1.52 (1.21-1.91)	<.001	0.68 (0.37-1.25)	.21	1.11 (0.96-1.29)	.15
	7 to 12 months	1.30 (0.90-1.88)	.17	1.52 (0.75-3.09)	.25	1.34 (1.03-1.73)	.026
	13 to 48 months	1.54 (1.30-1.82)	<.001	1.05 (0.67-1.65)	.83	1.62 (1.34-1.96)	<.001
	> 48 months	1.01 (0.83-1.24)	.89	1.18 (0.64-2.17)	.59	1.39 (1.11-1.75)	.004
Depression	Unexposed	REF		REF		REF	
	1 to 6 months	1.53 (1.28-1.82)	<.001	1.35 (0.87-2.08)	.17	1.70 (1.57-1.84)	<.001
	7 to 12 months	1.05 (0.75-1.46)	.78	1.18 (0.61-2.27)	.62	1.77 (1.51-2.08)	<.001
	13 to 48 months	1.25 (1.07-1.46)	.005	1.72 (1.20-2.46)	.003	1.43 (1.25-1.64)	<.001
	> 48 months	1.22 (1.00-1.50)	.04	1.52 (0.89-2.61)	.12	1.58 (1.30-1.93)	<.001
Suicide	Unexposed	REF		REF		REF	

© 2022 Garcia-Argibay M et al. *JAMA Network Open.*

	1 to 6 months	1.24 (0.79-1.95)	.35	1.19 (0.41-3.43)	.74	1.34 (1.10-1.64)	.004
	7 to 12 months	0.43 (0.13-1.46)	.18	1.06 (0.28-4.03)	.94	1.67 (1.14-2.44)	.008
	13 to 48 months	1.05 (0.74-1.49)	.78	0.61 (0.13-2.80)	.53	1.25 (0.9-1.737)	.18
	> 48 months	1.22 (0.78-1.92)	.38	0.43 (0.10-1.98)	.28	0.91 (0.57-1.46)	.71

© 2022 Garcia-Argibay M et al. *JAMA Network Open.*

eMethods.

Exposure

Using the National Prescription Register (PDR), we retrieved information on dispensed finasteride and dutasteride (Anatomical Therapeutic Chemical [ATC] codes G04CB01 and G04CB02, respectively), date dispensed, daily doses per pack, and number of packages, and created two types of time-varying exposure variables. First, a categorical variable with 5 levels (one for each type of medication; unexposed, finasteride, dutasteride, alpha blockers, and a combination of 5ARIs and alpha blockers) was created, and participants were classified as unexposed —the reference category— until the first date of prescription and into one of the four drug categories thereafter. Second, a variable representing the time under exposure was created by calculating the number of months that the given drug was used, assuming the daily dose was taken as directed for the main indication. Treated periods were categorized into 1 to 6 months, 7-12 months, 13 to 48 months, and 49 months or longer. After treatment cessation, time under exposure was continued forward. We focused on individuals with a first prescription of finasteride or dutasteride from the age 50 onwards, and those with a history of use of these drugs were excluded. Out of 2 236 876 men, 122 193, 152 522, 114 259, 116 940, and 116 326 were excluded for having each outcome respectively before the start of follow-up, leading to a final sample size of 2 114 683, 2 084 354 , 2 122 617, 2 119 936, and 2 120 550 respectively.

In order to control for the potential effect of other medications used to treat BPH, alpha blockers such as alfuzosin, tamsulosin, terazosin, silodosin, and doxazosin (ATC G04CA01-G04CA04, C02CA04), alone or in combination with 5ARIs, were included. The use of alpha blockers was considered to serve also as a control exposure because these drugs are used for similar indications, but not expected to be associated with increased risk in our outcomes. Time under exposure to alpha blockers was calculated similarly to 5ARI drugs. When an individual took a 5ARI and alpha blockers together at any time during follow up, they were considered to be exposed to both drugs from the start of the second drug (i.e., ever-users of both during follow up), but time under exposure was calculated based on 5ARIs alone.

© 2022 Garcia-Argibay M et al. *JAMA Network Open.*

eAppendix.

Sensitivity analyses defining the date of the dementia diagnosis as three years before the date of diagnosis showed that, as in the main analysis, the overall risk for developing dementia was higher in those taking 5ARIs than unexposed individuals (eTable 5). 132 657 men were excluded for having dementia before the start of follow-up. When period-specific risk was investigated, the association became more consistent than the main analysis, and 5ARIs were associated with higher risk in all periods, compared with unexposed individuals (eTable 6). When we restricted analyses to those who started 5ARI treatment at least 4 months after the start of the PDR, patients under 5ARI treatment no longer showed a significant increased dementia risk compared to unexposed individuals overall and at any time under exposure (eTables 7 and 8).Analyses were rerun after performing propensity score matching. Our cohort comprised 1 280 157 individuals, of whom 1 019 704 were matched controls for 5ARI and alpha blocker users (see eTable 9 for cohort characteristics). Results showed a similar pattern of results by which the lack of a dose-response did not suggest a casual relationship between 5ARIs and all-cause dementia or its types.

© 2022 Garcia-Argibay M et al. *JAMA Network Open.*
