# Supplementary material for: Association of 5α-Reductase Inhibitors With Dementia, Depression, and Suicide
Source: JAMA Netw Open. 2022 Dec 22;5(12):e2248135. doi: 10.1001/jamanetworkopen.2022.48135 (PMC9857015; doi:10.1001/jamanetworkopen.2022.48135)
Supplement: Supplement 2. — Data Sharing Statement [file jamanetwopen-e2248135-s002.pdf]

Data Sharing Statement

Garcia-Argibay. Association of 5α-Reductase Inhibitors With Dementia, Depression, and Suicide. *JAMA Netw Open*. Published December 22, 2022.
doi:10.1001/jamanetworkopen.2022.48135

Data
Data available: No

Additional Information
Explanation for why data not available: The Public Access to Information and Secrecy Act in Sweden prohibits us from making individual level data publicly available. Researchers who are interested in replicating our work can apply for individual level data at Statistics Sweden: www.scb.se/en/services/guidance-for-researchers-and-universities/.
